# Right Stellate Ganglion Blockade as a Bridging Therapy Prior to Sympathectomy in a Hemodynamically Unstable Adolescent With Ventricular Storm Secondary to Congenital Long QT Syndrome

**DOI:** 10.1155/cria/9787071

**Published:** 2026-04-27

**Authors:** Pedro Gabriel Dotto, Ian Novy Quadri, Rafael de March Ronsoni, Marcelo Murilo Mejia

**Affiliations:** ^1^ Department of Anesthesiology, Hospital Infantil Dr. Jeser Amarante Faria, Joinville, Santa Catarina, Brazil; ^2^ Department of Cardiology, Hospital Infantil Dr. Jeser Amarante Faria, Joinville, Santa Catarina, Brazil

**Keywords:** autonomic nerve blockade, long QT syndrome, stellate ganglion, sympathectomy

## Abstract

Ventricular electrical storm is a life‐threatening emergency, especially in pediatric settings. The condition is largely mediated by heightened cardiac adrenergic tone and may be triggered by acquired conditions, while therapeutic options remain limited. We report the case of a 15‐year‐old male adolescent with profound functional impairment and congenital long QT syndrome admitted to a tertiary pediatric cardiology referral center in Southern Brazil for the treatment of aspiration pneumonia, who subsequently presented with refractory ventricular electrical storm during hospitalization. Despite optimized medical therapy in the intensive care unit, the patient became severely hemodynamically unstable and was deemed unsuitable for immediate definitive cardiac sympathectomy. A temporary autonomic modulation strategy using right stellate ganglion blockade was proposed. The intervention was successful in suppressing the electric storm, promoting hemodynamic stabilization, and serving as a bridging therapy until sympathectomy could be safely performed under more stable clinical conditions.

## 1. Introduction

Electrical storm constitutes a potentially life‐threatening emergency, especially in pediatric cardiology settings. Acute systemic infections, inflammatory states, or metabolic stress may act as powerful arrhythmic triggers by amplifying autonomic imbalance. Therapeutic options are limited, and clinical instability may preclude definitive interventions, contributing to the high morbidity and mortality associated with these malignant arrhythmias [1].

Inherited myocardial channelopathies occupy a central role in this clinical context, with congenital long QT syndrome (LQTS) representing a prototypical condition. The syndrome was first described in the 1950s by Jervell and Lange–Nielsen through meticulous clinical observation of a single family in which four siblings presented with congenital deafness, prolonged QT interval, recurrent syncope, and sudden cardiac death. In an era preceding molecular genetics and modern electrophysiological tools, the authors demonstrated the hereditary nature of the condition by integrating family history, clinical manifestations, electrocardiographic findings, and postmortem observations. This refined, multidisciplinary clinical reasoning allowed the recognition of a novel arrhythmogenic entity and laid the foundation for understanding LQTS as an inherited disorder with malignant ventricular arrhythmias as its defining feature [2–4].

Advances in clinical electrophysiology and genetics have established LQTS as a major cause of sudden cardiac death in young individuals, emphasizing the need for early recognition and individualized management strategies [5, 6]. Subsequent experimental and clinical electrophysiological studies expanded this conceptual framework by identifying autonomic imbalance as a key mechanism in arrhythmogenesis. In particular, cardiac adrenergic tone originating from the stellate ganglion was shown to play a central role in modulating ventricular electrophysiology, acting as a trigger for malignant ventricular events in susceptible patients. These observations provided the physiological basis for antiadrenergic therapeutic strategies aimed at reducing arrhythmic burden in high‐risk individuals [7].

With the establishment of the International LQTS and advances in molecular genetics, multiple LQTS genotypes were identified, enabling more precise genotype–phenotype correlations and improved risk stratification [5, 6, 8]. The most prevalent subtypes, LQT1 and LQT2, result from loss‐of‐function mutations in potassium channel genes (Kv7.1 and Kv11.1, respectively), leading to reduced outward potassium currents during Phase 3 of the cardiac action potential and delayed repolarization. In contrast, LQT3 is caused by gain‐of‐function mutations in the cardiac sodium channel (Nav1.5), resulting in persistent late sodium current. In this context, sympathetic activation may precipitate a temporal mismatch between depolarization and repolarization, facilitating R‐on‐T phenomena and the initiation of malignant ventricular arrhythmias [3, 8].

Current management of LQTS focuses on antiadrenergic strategies, including β‐blocker therapy, avoidance of QT‐prolonging medications, and surgical cardiac sympathectomy. Implantable cardioverter‐defibrillators (ICDs) are frequently indicated in patients with prior resuscitated cardiac arrest or recurrent malignant ventricular arrhythmias despite optimized medical therapy [3, 4]. However, in patients presenting with ventricular electrical storm and severe hemodynamic instability, immediate surgical sympathectomy may not be feasible.

The stellate ganglion, formed by the fusion of the inferior cervical and first thoracic sympathetic ganglia, constitutes a major relay of cardiac sympathetic innervation. Although both stellate ganglia contribute to autonomic control of the heart, experimental and clinical data demonstrate lateralized effects on cardiac electrophysiology. The left stellate ganglion has been more strongly associated with ventricular repolarization and arrhythmogenesis, forming the anatomical basis for left cardiac sympathetic denervation as the standard surgical approach. Nevertheless, right‐sided sympathetic fibers also exert significant influence on cardiac electrophysiology, including ventricular excitability and arrhythmic susceptibility [9, 10].

In critically ill patients, anatomical constraints, concurrent neurological conditions, or device‐related limitations may restrict the feasibility of left‐sided interventions. In such scenarios, right stellate ganglion blockade (SGB) may represent a practical and effective alternative, allowing rapid attenuation of cardiac sympathetic drive while minimizing procedural risk. Clinical studies and systematic reviews have demonstrated that SGB can suppress refractory ventricular arrhythmias and electrical storm and may serve as a temporary antiadrenergic strategy bridging patients to definitive therapies, including surgical sympathectomy [10–12]. In pediatric patients, careful consideration of anesthetic dosing, pharmacological interactions, and procedural safety is essential, but available evidence supports the feasibility of this approach when performed in experienced centers [13].

## 2. Case Presentation

This case was conducted at Hospital Infantil Dr. Jeser Amarante Faria, Joinville, Santa Catarina, Brazil, from March 2024 to August 2025 and reported in accordance with the Enhancing the Quality and Transparency of Health Research (EQUATOR) guidelines. Written informed consent for publication was obtained from the patient’s legal guardian in accordance with the ethical principles of the Declaration of Helsinki and Brazilian legislation (National Health Council Resolution 466/12), following approval by the local research ethics committee (registration number 85912625.5.0000.5363). Clinical data were extracted from the patient’s medical records.

**FIGURE 1:**
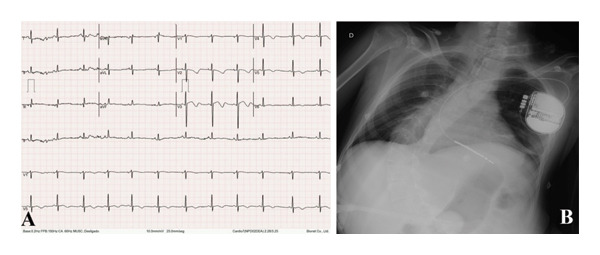
(A) Electrocardiogram recorded under propranolol and propafenone daily use, showing a sinus rhythm, cardiac frequency of 72 bpm, abnormal left ventricular repolarizations, and a prolonged QT interval of 480 ms (Bazett’s correction = 526 ms). (B) Posteroanterior chest X‐ray exhibiting proper positioning of the ICD, gastrostomy feeding tube, tracheostomy, and accentuated scoliosis.

**FIGURE 2:**
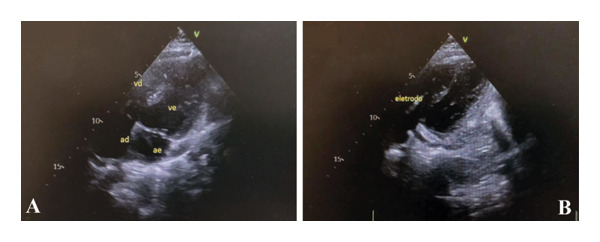
Transthoracic echocardiogram. (A) Apical four‐chamber view demonstrating *situs solitus* with atrioventricular concordance, obtained in the late postoperative period after repair of tetralogy of Fallot, with no residual structural lesions. (B) Apical view highlighting the position of ICD lead.

The patient is a male adolescent with severe neurological disability and marked underweight, in whom congenital LQTS was diagnosed later in life. He was born at term with tetralogy of Fallot, surgically corrected at 10 months of age, and subsequently developed epilepsy. At 8 years of age, he suffered a resuscitated cardiac arrest lasting approximately 10 min, resulting in hypoxic–ischemic encephalopathy with spastic tetraparesis, aphasia, and complete dependence for activities of daily living, requiring gastrostomy for nutritional support. Genetic testing later identified a familial *KCNH2* mutation, confirming LQTS type 2, and an ICD was placed (Figures 1 and 2). Long‐term therapy included phenytoin (5 mg/kg/day) and propranolol (2 mg/kg/day).

At 15 years of age, weighing 33 kg, the patient was admitted to the hospital with aspiration pneumonia. A few hours after admission, he developed an acute episode of hypoxemia associated with bradycardia, requiring endotracheal intubation and transfer to the intensive care unit (ICU). Rapid‐sequence induction was performed using midazolam (0.2 mg/kg), ketamine (2 mg/kg), and atropine (10 μg/kg). Continuous sedation was maintained with midazolam (0.1–0.3 mg/kg/h) and fentanyl (1–3 μg/kg/h). Due to the prolonged use of endotracheal tube, tracheostomy was indicated.

After resolution of the pulmonary infection and initiation of sedation weaning, the patient developed recurrent episodes of torsades de pointes and ventricular fibrillation, each progressing to cardiac arrest. All events were managed according to the American Heart Association Pediatric Advanced Life Support guidelines. The ICD delivered appropriate shock therapy during these episodes. In total, 17 effective ICD shocks were delivered, and the cumulative duration of cardiac arrest was 18 min. Propranolol (5 mg/kg/day), magnesium sulfate (0.5 mEq/kg/day), and continuous lidocaine infusion (20 μg/kg/min) were administered. Electrolyte levels were closely monitored and remained within normal ranges, and QT‐prolonging medications were strictly avoided. As isoproterenol was unavailable, a temporary transvenous pacemaker was inserted for heart rate control.

In this context, cardiac sympathectomy was indicated. However, due to profound hemodynamic instability, the patient was deemed unsuitable for immediate surgical intervention. SGB was therefore considered as a temporary strategy to improve autonomic balance and hemodynamic stability. A right SGB was performed under ultrasound guidance, as the left side was occupied by a central venous catheter. The side selected for the catheter placement was previously determined by the anatomical feasibility. A total of 8 mL of 0.5% ropivacaine was administered. Lidocaine infusion was discontinued right before the procedure. No procedural complications were observed.

Immediately after the procedure, a progressive reduction in arrhythmic episodes was observed over the following hours, and electrical instability resolved within 24 h following the procedure, allowing successful extubation. Fourteen days later, left cardiac sympathectomy was performed without complications, and the patient was discharged from the ICU on the same day. At that stage, a unilateral procedure was preferred in order to minimize surgical stress, given the patient’s markedly debilitated state. Hospital discharge occurred 5 days thereafter. Three months later, after further clinical recovery, the right cardiac sympathectomy was scheduled and subsequently performed under safer conditions, and it was also completed uneventfully.

After almost 2 years, the patient remains under outpatient follow‐up, with sustained clinical and cardiologic stability. Maintenance therapy includes propranolol (2 mg/kg/day), propafenone (10 mg/kg/day), furosemide (1 mg/kg/day), and phenobarbital (6 mg/kg/day).

## 3. Discussion

This report highlights SGB as a potential life‐saving rescue strategy for critically ill pediatric patients with malignant ventricular arrhythmias. In such scenarios, therapeutic options are limited, and clinical instability may preclude immediate definitive interventions. The present case shares important similarities with prior reports, particularly the use of SGB as a bridging therapy following failure of optimized pharmacological and device‐based interventions.

Metabolic or physiological perturbations may be sufficient to trigger malignant arrhythmias in predisposed patients, reflecting a reduced arrhythmic threshold inherent to conditions such as congenital LQTS. Systemic infections, ventilatory weaning, and critical illness may amplify adrenergic tone and precipitate refractory ventricular electrical storm, even in the presence of optimized pharmacological therapy and ICD support, as illustrated herein [1, 3, 4].

Our patient was an older adolescent with complex congenital heart disease, severe neurological impairment, and a late diagnosis of LQTS, who developed ventricular electrical storm in the setting of pneumonia and adrenergic activation during ventilatory weaning. These features reinforce the role of acute systemic illness as a powerful arrhythmic trigger in genetically predisposed patients.

The stellate ganglion represents a major relay of cardiac sympathetic innervation, receiving preganglionic fibers from the thoracic spinal cord and modulating ventricular electrophysiology through postganglionic cardiac projections [9]. Experimental and clinical electrophysiological studies have demonstrated that autonomic imbalance plays a central role in arrhythmogenesis, acting either as a primary driver of electrical instability or as a trigger for malignant ventricular events in genetically susceptible patients [7].

Based on this physiological rationale, left cardiac sympathetic denervation has become an established therapy for patients with refractory ventricular arrhythmias in LQTS, particularly when pharmacological strategies fail [3, 8]. However, surgical sympathectomy may not be immediately feasible in critically ill patients due to hemodynamic instability, active infection, or competing clinical priorities, highlighting the need for temporary and less invasive autonomic modulation strategies.

In this context, SGB has emerged as an effective rescue therapy for ventricular electrical storm in adult populations. Systematic reviews have demonstrated high rates of arrhythmia suppression following SGB, with most reported cases involving left‐sided blockade in middle‐aged or elderly patients [11, 12]. Pediatric experience with SGB, however, remains scarce. One of the earliest pediatric reports described a 6‐year‐old child with familial LQTS in whom left SGB successfully controlled recurrent malignant arrhythmias and allowed subsequent surgical sympathectomy [13].

A particularly novel aspect of this case is the use of right‐sided SGB. Although left‐sided blockade is generally preferred due to its greater influence on ventricular repolarization, anatomical constraints related to the presence of a left‐sided central venous catheter precluded its use. Notably, right SGB resulted in rapid and sustained suppression of malignant ventricular arrhythmias, supporting the functional relevance of right‐sided cardiac sympathetic pathways. Previous adult series reported left‐sided blockade in the majority of cases, with exclusive right‐sided blockade rarely described [11, 12]. This observation suggests that lateral preference should not preclude SGB when clinical circumstances limit access to the left side. In this case, the left side was selected for central venous catheter placement due to anatomical feasibility, as the patient was severely debilitated and had a history of prior cardiac surgery and prolonged stay in the ICU. However, whenever possible, left‐sided vascular access should be avoided in patients with LQTS in order to preserve the preferred side for SGB.

Dosing and safety considerations are particularly important in pediatric patients. Standardized SGB dosing protocols for children are lacking, and most recommendations are extrapolated from adult data [10]. Pediatric patients are at increased risk of local anesthetic systemic toxicity due to lower body mass, immature hepatic metabolism, and higher free drug fractions [14]. In critically ill patients, additional risk arises from pharmacological interactions, especially with antiarrhythmic agents [15, 16]. In our case, 8 mL of 0.5% ropivacaine was administered to a 33‐kg adolescent who was also receiving lidocaine infusion. Although this dose approached the upper safety threshold, it was selected to maximize the likelihood of effective sympathetic blockade in a life‐threatening situation where repeat procedures were impractical. Lidocaine infusion was paused right before the procedure, and lipid emulsion therapy was available at the hospital. No clinical signs of local anesthetic systemic toxicity were observed. Nevertheless, SGB is not without risk, as the stellate ganglion lies in close proximity to critical vascular and neural structures. A systematic review identified hoarseness and cervical hematoma as the most frequently reported complications following SGB between 1990 and 2018 (Table 1) [17].

**TABLE 1:** The most frequently reported complications following stellate ganglion blockade between 1990 and 2018 (adapted from Goel et al. [17]).

Complication	Pathophysiological mechanism	*N* (%)
Hoarseness	Recurrent laryngeal nerve paralysis and unilateral vocal fold paresis	73 (28.1)
Hematoma	Inadvertent vascular puncture	41 (15.8)
Light‐headedness	Sympatholytic vasodilation with transient reduction in peripheral vascular resistance or systemic absorption of the local anesthetic	20 (7.7)
Blood aspiration	Inadvertent vascular puncture	20 (7.7)
Hypertension	Local anesthetic spread along the carotid sheath resulting in vagal block	13 (5.0)
Brachial plexus block	Spread of the local anesthetic to the brachial plexus, resulting in ipsilateral motor and sensory blockade of the upper limb	12 (4.6)
Dysphagia	Blockade of the glossopharyngeal, vagus, or superior laryngeal nerves, or local edema compressing pharyngeal structures	11 (4.2)
Cough	Direct tracheal irritation, partial anesthesia of the superior laryngeal nerve, or microaspiration due to dysphagia	7 (2.7)
Intrathoracic bleeding	Inadvertent vascular puncture	7 (2.7)
Subdural or intraspinal blockade	Inadvertent injection of local anesthetics into the subdural, epidural, or subarachnoid space	5 (1.9)
Seizures	Inadvertent vascular injection of local anesthetics	5 (1.9)
Transient locked‐in syndrome	Inadvertent vascular injection of local anesthetics and transient anesthesia of the brainstem	4 (1.5)
Persistent ptosis	Blockade of the ocular sympathetic fibers	3 (1.2)
Pneumothorax	Inadvertent apical pleural puncture	3 (1.2)
Contralateral Horner’s syndrome	Contralateral spread of the local anesthetic into the prevertebral or cervical epidural space, resulting in blockade of sympathetic fibers on the opposite side	2 (0.8)
Total of complications following Stellate Ganglion Blockade reported between 1990 and 2018		**260 (100)**

*Note:* The bold value represents the full account of the reported complications.

Assessment of block success in sedated or critically ill patients may be challenging, as classical indicators such as Horner’s syndrome or skin temperature changes may be attenuated or absent [10]. In the present report, effectiveness was confirmed by the rapid resolution of ventricular arrhythmias within 24 h, hemodynamic stabilization, and successful extubation. Definitive surgical sympathectomy was subsequently performed under safer clinical conditions, with sustained arrhythmic control during follow‐up.

This report has several limitations inherent to its design. As a single‐patient case report, it does not allow causal inference or generalization to the broader pediatric population with congenital LQTS. The clinical course occurred in a complex critical care setting, with multiple potential confounding factors, including systemic infection, mechanical ventilation, sedation, and concurrent antiarrhythmic therapies, making it difficult to attribute arrhythmia suppression solely to SGB. Objective markers of successful sympathetic blockade, such as Horner’s syndrome or serial perfusion indices, were not systematically documented, and efficacy was primarily inferred from temporal clinical improvement. Additionally, right‐sided SGB was performed due to anatomical constraints, a strategy that remains less well studied than left‐sided blockade. Finally, although no adverse events were observed, the local anesthetic dose approached the upper safety threshold.

In conclusion, when pharmacological and device‐based therapies fail and immediate sympathectomy is not feasible, SGB may provide effective temporary autonomic modulation, even when right‐sided blockade is required due to anatomical or clinical constraints. Longer follow‐up and larger studies are required to better define the safety, optimal dosing, and reproducibility of this approach in autonomic modulation in children.

## Funding

No funding was received for this research.

## Conflicts of Interest

The authors declare no conflicts of interest.

## Data Availability

All clinical data relating to this study can be found in the hospital where it was conducted and can be made available upon request when the requests are properly justified.
